# Experiences of women with disabilities during and after COVID-19: Needs, sources of support and implications for policy and practice

**DOI:** 10.1371/journal.pone.0342900

**Published:** 2026-02-23

**Authors:** Adi Finkelstein, Sara Genut, Anat Golos

**Affiliations:** 1 Selma Jelinek School of Nursing, Faculty of Life and Health Sciences, Jerusalem College of Technology, Jerusalem, Israel; 2 Department of Bioinformatics, Faculty of Life and Health Sciences, Jerusalem College of Technology, Jerusalem, Israel; 3 School of Occupational Therapy, The Faculty of Medicine, The Hebrew University, Jerusalem, Israel; Neighborhood Physical Therapy, UNITED STATES OF AMERICA

## Abstract

Approximately 700 million women and girls worldwide live with disabilities, facing compounded discrimination due to both gender and disability. Despite their large numbers, they are often excluded from decision-making and rendered invisible. Research shows that crisis such as nature disasters and public health crises affects people with disabilities more severely than the general population, and women more than men. Yet, studies focusing specifically on women with disabilities in such contexts remain scarce. The present qualitative study aimed to address this gap by analyzing 56 semi-structured interviews with women with motor and/or sensory impairments about their life experiences during COVID-19 and afterwards, using reflexive thematic analysis. Participants aged 26–69, mostly with motor impairments (66.1%), reported satisfaction with their health (71.4%). Findings are organized into four main themes and six sub-themes corresponding to the components of the International Classification of Functioning, Disability, and Health (ICF) as follows: Physical and mental health through daily care and environmental contexts; Navigating accessibility and attitudinal barriers in daily life; Maintaining decision-making autonomy and financial independence; Family and social relationships as sources of support. The findings demonstrate multifaceted impacts on women with disabilities across personal, social, and contextual domains, during both stable periods and crises. To effectively address their needs, policymakers should incorporate the perspectives of women with disabilities in the planning of support programs for both routine periods and crises responses.

## Introduction

The Convention on the Rights of Persons with Disabilities (CRPD), adopted by the United Nations (UN) General Assembly on December 13, 2006 [1], declared that: “States Parties shall take all appropriate measures to ensure the full development, advancement and empowerment of women, for the purpose of guaranteeing them the exercise and enjoyment of the human rights and fundamental freedoms set out in the present Convention” (Article 6).

Globally, it is estimated that around 700 million women and girls live with disabilities. According to the UN Women, the average rate of disability among females is 19.2%, compared to 12% among males, indicating that approximately one in five women is affected by disability. Women with disabilities face unique challenges and are subject to double discrimination stemming from both their gender and their disability [2].

Despite their substantial numbers, women and girls with disabilities are often rendered invisible and excluded from the decision-making processes that shape their lives [3]. Matin et al. [4] found that women with disabilities encounter multiple barriers when attempting to access healthcare. These barriers include negative attitudes, low health literacy, inadequate insurance coverage, inaccessible medical equipment, and communication barriers. Women with disabilities also face an exceptionally high risk of physical, sexual, and emotional abuse, specifically related to their disability [5]. Monedero et al. [6] highlighted that women and girls with disabilities continue to face significant barriers to education, despite progress toward inclusive social models. Many are excluded from educational systems and vocational training and often lack basic literacy opportunities. These and other factors contribute to the poverty and social exclusion experienced by women with disabilities.

Given their reliance on services and caregivers to meet specific needs, along with their potential for increased susceptibility to illness, people with disabilities are considered particularly vulnerable to nature disasters [7–10] and during public health crises [11,12] (hereafter: crisis). Such crises often exacerbate long-standing inequities and intensify existing structural disadvantages faced by people with disabilities. Research further demonstrates that these barriers are socially and systemically constructed, rather than inherent to disability itself [13,14]. The COVID-19 pandemic drastically impacted the physical, social, and emotional condition of people with disabilities [11,15,16]. The pandemic significantly disrupted social support and inclusion for people with disabilities [17], who already experienced generally higher levels of social isolation and loneliness compared to those with no disability [11,12,18,19].

Women with disabilities are more vulnerable than men, particularly in crisis, due to the intersection of gender-based and disability-based discrimination. They may encounter significant barriers in accessing information, mobility, and support systems, which increase their dependence on others [20,21]. Comparative international assessments demonstrate reduced access to healthcare, increased exposure to violence, and the weakening of support networks across diverse socio-cultural contexts [22,23]. In a study from the United States [24], fourteen women with disabilities reported that following the COVID-19 pandemic, they experienced worsening or newly developed mental health symptoms, along with limited access to healthcare and shopping services. A study from South Africa [25] found that young women with disabilities experienced compounded livelihood disruptions during the pandemic, illustrating how gender and disability intersect to shape crisis experiences. Evidence from Canada shows that even within high-income welfare states, women with disabilities experienced intensified caregiving responsibilities, economic precarity, and social isolation, revealing the persistence of systemic inequities [26,27]. Likewise, findings from Turkey emphasize the continuing health challenges and gendered inequalities faced by women with disabilities during the COVID-19 pandemic [28]. Collectively, these cross-regional findings underscore the need to examine how women with disabilities actively navigate and adapt to changing conditions during and after crises.

### Study aim

This qualitative study examines the experiences of women with disabilities in Israel during and after the COVID‑19 pandemic. Using the International Classification of Functioning, Disability, and Health (ICF) [29], as our analytical frame, we analyze how body functions, social structures, and environmental factors interact to shape these experiences across periods of stability and crisis. This information is essential for developing targeted policies and programs that effectively tackle the unique challenges faced by this population [4,30].

### Theoretical framework of the study

The ICF [29] is grounded in the biopsychosocial approach to disability. The model views disability as a complex interaction between health conditions and contextual factors, both environmental (e.g., social attitudes, accessibility, and legal structures) and personal (e.g., age, gender, education, and coping style). It includes two main lists: body functions and structures, and domains of activity and participation. *Functioning* refers to all aspects of body function, activities, and participation, while *disability* encompasses impairments, activity limitations, and participation restrictions. The model also considers environmental factors that influence these components.

The ICF framework defines the participation of people with disabilities as involvement in life situations that occur within the familial, social, and occupational environments to which an individual belongs, considering their health condition [31]. It uses two main qualifiers to assess Activity and Participation. The Performance qualifier and the Capacity qualifier describe an individual’s ability to execute a task or an action. The Performance qualifier captures the life experience: what a person does in their current environment, including the use of assistive devices or personal support. It reflects real-world functioning and involvement in daily life.

### Study background

The State of Israel signed the Convention on the Rights of Persons with Disabilities (CRPD) in 2007 and ratified it in 2012. In 2022, approximately 1.7 million individuals in Israel were recognized by state institutions as persons with disabilities (including children under the age of 18). About half of the persons with disabilities aged 20–64 were women [32]. However, women with disabilities in Israel are particularly marginalized in the areas of education, employment, and health. Twenty percent of women with disabilities achieve higher education, compared to 16% of men with disabilities. However, employment rates reveal stark disparities: only 35% of women with severe disabilities and 51% with moderate disabilities are employed, versus 74% of women without disabilities. For men, the rates are 40% for severe and 69% for moderate disabilities, while 84% of men without disabilities are employed. Healthcare access is also a challenge, as clinics are often physically inaccessible and lack gender-sensitive services, disproportionately affecting women with disabilities. Public authorities in Israel do not collect specific data or systematically address the unique barriers faced by women with disabilities, hindering targeted service development and potentially reinforcing stigmatization in public services [33,34].

## Materials and methods

### Study design

Adopting a phenomenological orientation [35], we conducted a cross-sectional qualitative study using retrospective, semi-structured interviews.

### Data collection

To meet the inclusion criteria, the participant had to be a woman over the age of 18 years with self-reported motor impairments and/or sensory impairments (visual and/or hearing) that interfere with her functioning in daily activities. The exclusion criteria were reporting cognitive and/or emotional-mental impairment in addition to the above-mentioned impairments. This decision was made to maintain a higher level of homogeneity in the research study group as is commonly recommended in phenomenology oriented qualitative research in order to reach saturation [36]. According to Morse [37], indicators of saturation and data richness become apparent as the researcher develops greater familiarity and competence with the study topic. Due to the paucity of existing data on the study aim, the research team decided to include all women who expressed a willingness to participate, even after data saturation had been reached.

The data were collected between April 1 and July 30, 2022. Participants were recruited by research assistants using a convenience sample, based on personal acquaintances, either face-to-face or by phone. Women who showed interest in participating in the study received an oral explanation about the research goals; upon meeting the inclusion criteria, they received a detailed information sheet about the research, its purposes, and about the research team, and signed a written informed consent form.

### Characteristics of the participants

The study included 56 women with disabilities. All the participants were Jewish women, aged 26–69 years (*M* = 43.06, SD = 12.90), who lived in Israel. Most of them identified themselves as religious. As seen in Table 1, Most participants (66.1%, n = 37) reported physical-motor impairment (hereafter: physical-motor); some of the participants reported sensory impairments (visual: 19.6%, n = 11 and/or hearing: 23.2%, n = 13). Most of them reported having professional or academic degrees (67.9%, n = 38). A majority expressed satisfaction with their health (71.4%, n = 40). Approximately half of the sample (53.6%, n = 30) reported having children, with nearly one-third of them (28.6%, n = 16) indicating that they had between four and seven children.

**Table 1:** Socio-demographic characteristics of the participants (n = 56).

	n	%
Impairment		
Physical-motor		
Yes	37	66.1
No	19	33.9
Sensory (visual)		
Yes	11	19.6
No	45	80.4
Sensory (hearing)		
Yes	13	23.2
No	43	76.8
Level of religiosity		
Secular	9	16.1
Religious	43	76. 8
Missing	4	7.1
Family status		
Living alone	27	48.2
Living with family members	29	51.8
Having children		
No	26	46.4
Yes	30	53.6
1–3 children	14	25.0
4–5 children	11	19.7
7 children	5	8.9
Residence		
City	45	80.4
Other	11	19.6
Education High school or less	18	32.1
Professional or academic degree	38	67.9
Employed		
No	20	35.7
Yes	35	62.5
Missing	1	1.8
Education or caregiving sector	13	23.2
Office setting	7	12.5
Other	15	26.7
Income		
Lower than the monthly minimum wage	27	48.2
About the monthly minimum wage	6	10.7
Higher than the monthly minimum wage	23	41.1
Perceived Health Satisfaction		
Very dissatisfied/ Dissatisfied	16	28.6
Somewhat satisfied/ Satisfied/ Very satisfied	40	71.4

### Instruments

Semi-structured individual interviews were conducted with a few leading questions, where participants were asked to describe their life experiences across various areas, such as work, family, education, social life and more, their needs and sources of support. Participants were asked to reflect on these issues in retrospect, considering their experiences during the time of the COVID-19 pandemic. Interviewers were instructed to allow the conversation to “flow” according to the interviewee’s preferences.

### Procedure

The interviews were conducted by the research assistants via Zoom or face-to-face, according to the participant’s preference; they lasted an average of one hour. All the interviews followed the same guide. Only the participants and interviewers were present at the interview. The interviews were audio-recorded and transcribed verbatim by the research assistants. Transcripts were checked against audio recordings for accuracy by the first author. The first author assigned a random number to each transcript to ensure participant anonymity.

The research assistants received detailed instructions from the first and second co-authors before commencing the fieldwork regarding the study’s aims. Their preparation included training in conducting qualitative interviews and an online course in research ethics. Ongoing reflexive discussions between the first co-author and the research assistants accompanied the fieldwork. The first co-author took field notes.

We did not conduct follow-up interviews but encouraged participants to contact us if they wanted to add anything to their original statements. By the end of the fieldwork, none of the participants had reached out to us. We offered participants the opportunity to review the interview transcripts; however, none of them wished to do so. Several participants expressed interest in receiving the final published results of the study.

### Analysis

Qualitative data were manually analyzed without the aid of software or AI following reflexive thematic analysis principles [38,39], with the guidelines of Clarke and Bruan [40]. The analysis begun inductively in collaboration among the three co-authors, with the first author taking the lead. After Phase 1 (familiarization with the data), we continued by generating initial codes (Phase 2). An open coding of 15 randomly selected transcripts was conducted simultaneously by the three co-authors. Following several discussions, the remaining 41 transcripts were divided among the three co-authors. Each continued coding her transcripts, and after several discussions, we began developing themes (Phase 3). Consistent with reflexive thematic analysis [38], the aim of coding was to enhance reflexivity and interpretative depth rather than to achieve coder consensus. The process resulted from deep, prolonged engagement with the data. Through this process, we identified a strong correspondence between the themes and specific components of the ICF model. Therefore, we decided to continue by conceptualizing the themes deductively. Within reflexive thematic analysis, a deductive stance entails interpreting data through a pre‑existing theoretical lens, rather than testing that theory or specific hypotheses [38]. The first co-author completed Phases 4–6 and produced the final report, which was discussed and approved by the other co-authors.

### Rigor and reflexivity

We followed the guidelines for Consolidated Criteria for Reporting Qualitative Research [41]. All three authors are women with PhDs who serve as lecturers and researchers in academic institutions. The first author is a medical anthropologist with extensive experience in qualitative health research, including a diverse body of research in disability studies; the second is a chemistry lecturer with a strong background in science education; and the third is an occupational therapist affiliated with Participation in the Community and Environment (Socio-Cultural) Research Laboratory. The research team had no personal or professional involvement in the topic and held no prior stance on the findings. Engaging multiple researchers from diverse disciplines contributed to the understanding of the data by offering a deeper perspective on the research question, helping to uncover essential details and nuances, and promoting consistent interpretation of the findings [42].

### Ethics

This study was approved by the Ethics Committee of the Jerusalem College of Technology (No. 006_21).

## Results

In the analysis we integrated four main themes and six sub-themes describing the lived experiences of women with disabilities in COVID-19 pandemic and afterwards:

### 1. Physical and mental health through daily care and environmental contexts

#### 1.1 Embodied experiences of physical health across changing environments.

The participants placed great importance on their health condition as a central aspect of their lived experience: “I repeat – only health! Quality of life is health” (A2- motor). They shared how they maintained their physical condition daily, including medical follow-ups and tests, nutrition, and other treatments such as hydrotherapy and physiotherapy: “Of course, sports and... food, you maintain your diet, so you contribute to your health” (C3- motor).

The participants referred to their body functions as a main component influencing their health. They described how these functions are shaped by the broader environmental context in which individuals live. A few participants with motor disability recalled improvement in their health during the outbreak of the COVID-19 pandemic. They did not leave their house, reducing their physical effort in coping with everyday life, and they could rest when needed:

… following the COVID-19 pandemic, …. I didn’t work and I felt that my body was resting…. My back pains improved because I rested.... It was winter... and when I’m outside at this time of the year, I suffer more pain because of my back. At home, I had heating, so I felt these pains less. (R1- motor)

However, others reported negative consequences of the lockdowns on their health. For example, women with motor and breathing difficulties reported challenges in carrying out regular treatments and medical visits; and as a result, their health condition deteriorated:

I couldn’t go outside and had to stop activities related to my health condition, like sports.... Many tests were postponed.... Medical follow-up was much less frequent... These are, of course, things that are very, very weakening. (E1- motor)

Participants’ accounts show careful attention to bodily functions such as mobility, pain, and endurance, which they experienced as central to their well-being.

#### 1.2 Mental health and its interplay with life meaning, satisfaction, and religious faith.

Topics such as life goal fulfillment, joy of life, and overall life satisfaction were identified as important components of the participants mental health and well-being: “ … I do suffer from pain in my neck and lower back... but I am a happy woman in general. I am happy with my family…. I love my job…” (R1-motor); “... being healthy [is important], having the strength to get up and do all the things I need to do; but it’s also really important to live with joy” (T2- hearing).

Many emphasized that their religious faith was a key source of mental strength, particularly during the pandemic. They highlighted religious practices as a fundamental part of their daily lives. Belonging to a religious community provided them with support and strength both in daily life and during the pandemic: “Prayers, observance of mitzvot [actions performed by religious people], study and memorization of [religious] laws and morals, etc., – it fills me up... and gives a great deal of satisfaction” (B2-visual and hearing). Participants’ experiences indicate that religious practice helped reinforce emotional resilience, stability, and a sense of continuity during the pandemic. The communal dimensions of religious life further provided a collective source of support, offering social connectedness and a shared identity that mitigated feelings of isolation and uncertainty.

### 2. Navigating accessibility and attitudinal barriers in daily life

Participants described how inaccessible environments significantly impacted their daily life experiences, requiring careful planning for nearly every outing and sometimes forcing them to modify or abandon their plans altogether: “There is no such thing as spontaneity.... I always must find out about everything beforehand... to know that there is accessibility everywhere, that there are stairs, that there are accessible transportation and accessible roads to all places” (D1- motor).

Participants recalled that managing day-to-day life from home during the pandemic made life easier because it spared them the frustration of confronting the inaccessible physical environment. Their adapted home environment acted as a supportive setting that minimized daily obstacles and momentarily transformed participants’ lived experience of disability. As one participant described:

During the pandemic, I was at home most of the time…. In the few times I left home, [it stood out] how inaccessible the physical environment is for me…. [My] home is completely suited to my needs; I feel I almost have no disability…. To remind you, I am paralyzed in four limbs. (K2- motor)

Participants noted that online technologies supported their continued engagement in daily activities. Online services such as shopping, payments, and administrative tasks with banks and government offices expanded significantly during the pandemic, enhancing participants’ autonomy and participation, both during crises and beyond: “Before [the pandemic], I had daily difficulties when I had to travel, in terms of physical inaccessibility… but now because of the pandemic everything is online. So, it is much easier” (N1- motor).

It became a little more convenient... instead of going and submitting forms, they send it to you by email, you sign and return it by email, and then you don’t have to go there yourself…. It’s good, because... it takes the load off. (B3- motor)

A few participants shared that the improvement of online services, and having more free time during the pandemic, allowed them to realize personal dreams and ambitions. Two interviewees wrote a book:

I was at my parents’ house for a month and had nothing to do. I had the computer for two hours a day, so I sat and wrote about my disability... I found a publisher.... By September I already had a book in hand. (N2- visual)

Two participants studied online courses: “I learned coaching and developed myself…. I found a venture that I love so much” (N3- visual).

One participant pointed out that online meetings, where only her face was visible on the screen, sometimes help her to overcome prejudice attitudes because she passed as “normal” rather than quadriplegic:

On Zoom I don’t have to deal with how I think you perceive my paralyzed body…You don’t see me in a wheelchair. You see me as a person like everyone else... In face-to-face meetings, my disability becomes more present … [It is] impossible for others to ignore it. (K2- motor)

This example illustrates how virtual interaction can momentarily remove the visual cues that often shape discriminatory perceptions, enabling the participant control how she is seen and engaged on more equal terms, and showing how digital environments may mediate stigma by narrowing the gap between embodied difference and social evaluation.

However, participants with hearing impairment noted greater challenges in following meetings through online communication technologies, especially when there were many participants, and/or when they had difficulty seeing the speaker’s lips. One of the interviewees said that following the pandemic she decided to start wearing a hearing aid, something she had been ashamed to do in the past: “Now I have a hearing aid, which is easier for me, and I don’t have to be ashamed anymore” (A3- hearing).

### 3. Maintaining decision-making autonomy and financial independence

#### 3.1 Dependence and independence in daily life tasks and decisions.

The participants, to varying degrees, relied on support from their surroundings to carry out daily activities. They appreciate the help they receive as well as the people who provide it, recognizing that such assistance is essential for remaining active, participating in daily life, and pursuing their aspirations. At the same time, they describe this reliance as a constraint on their autonomy: “To be dependent on others, on their availability, on their kindness... it’s complicated because they’re not always there for me... and that’s something that stresses me out a lot and stops me from going out” (B2- visual). They described the ability to independently plan, organize, and manage time and space as a central aspect of their life experience, although they do not always succeed in achieving it: “… You need to be able to choose. To do what you want… not to be constantly dependent on others... It is above all... to be independent” (I3- motor).

These accounts highlight the ambivalence inherent in support relationships: assistance enables participation, yet the dependence it creates can generate uncertainty, limit spontaneity, and reinforce structural barriers to independence.

Participants’ reflections illustrate how crises can prompt a reassessment of one’s capabilities and dependencies, fostering a more proactive orientation toward autonomy and preparedness. They described how the pandemic disrupted their sense of security and control, with its sudden onset and existential threat underscoring their reliance on others and heightening vulnerability. In its aftermath, they emphasized the importance of reducing dependence during periods of stability to withstand future crises better: “I can say that the pandemic was a trigger….It [the pandemic] showed you that everything is out of control and that everything changes, and you have no control over it, and it’s up to you to deal with it” (C1- hearing).

I fired my caregiving assistant.... She continued to meet with her friends, and I couldn’t trust their level of protection from the virus... so, it was quite dramatic. It forced me mentally to learn to cope alone with what I can and somehow learn to manage with what I can’t handle. (E1- motor)

#### 3.2 Financial independence across variable life conditions.

All participants emphasized that financial independence and making a living are essential to them and that they are willing to make an effort to achieve it, even though it is not always easy for them to meet workplace expectations due to disability-related challenges. For this reason, some participants stated that they prefer to be self-employed rather than salaried employees: “The opportunity to go to work and make a living helps me keep my independence” (P1- motor); “My work and my workouts they’re really important to me… so I’ll have the strength to go out and give to others” (Q1- visual).

During the pandemic, participants were able to continue working from home thanks to digital technologies, thereby maintaining their financial stability. However, a few single participants also reported that economic strain forced them to give up their rented apartments and return to their parents’ homes. Participants who shoulder primary caregiving and household responsibilities faced growing economic hardship. One mother described that she struggled to provide her children with a daily hot meal, which they typically received as part of their educational program: “There was a lack of income and many expenses. The children usually eat lunch at kindergarten, but when they were home, I had to buy groceries and cook them, even though I did not work” (H1- motor).

These experiences show that the pandemic unevenly distributed economic risk and that limited income opportunities threatened participants with basic material security.

### 4. Family and social relationships as sources of support

#### 4.1 Nurturing family relationships and family care.

Participants emphasized the central role of close family in their lived experiences, describing it as a steady source of support. For participants who were mothers, their children were described as a source of pride and motivation.

Participants described both the challenges and the advantages of spending the pandemic at home with their families. Participants with young children highlighted the opportunity for meaningful family time: “We learned to be more patient and to enjoy the little things” (L1- motor); “We took out a guitar and sang, we prayed together…. There were lots of great moments with the children” (K1- visual); “We had a lot of time together and had fun… playing sports, cooking, and baking. It was a great time” (R1- motor). Others reported considerable stress stemming from the need to care for their children around the clock and manage increased household responsibilities: “The children were at home all the time. There was no fixed daily routine. How much can you keep four children busy at home, when the youngest is six and a half years old?” (K1-visual).

Participants who move back in with their parents also described in a positive light their quality time together: “I had an amazing time! I went back to living with my parents for a few months, after eight years of not living at home. We are much closer now” (N3- visual).

These examples show the dual nature of family life during crises. Family relationships can provide emotional grounding, yet they also intensify caregiving demands, especially for mothers with disabilities.

#### 4.2 Social relationships as supportive resources.

Participants reported that social interaction, whether at work, with friends, were significant elements of their lived experience: “I can cope with everything... but without my good friends it would be very difficult for me” (D2- motor); “Family and social environments have the most significant impact on mood and overall functioning in the end” (R2- motor).

Participants shared that since the pandemic, online communication technologies have enabled them to maintain and, in some cases, strengthen their social connections: “In normal times, I can’t go to visit my friends’ houses because they are not accessible. With Zoom I can see their houses... I can see their children…. This is a pleasure” (K2- motor). On the other hand, some participants reported feeling overwhelmed: “Because of ZOOM, I felt that there were too many people in my life. I was constantly in the company of people” (K2- motor).

Participants emphasized the importance of direct, face-to-face human interaction, which they found lacking during the pandemic. A participant with quadriplegia shared: “The pandemic didn’t change anything for me. I’m used to spending most of my time at home by myself. What I lacked mostly was guests” (B1-motor). A few participants shared that they experienced loneliness: “Physically, it [communication via Zoom] was easier for me, but mentally, being isolated from people had an impact on me” (R2- motor). “I felt like I was in prison. Although a prisoner in my home, still... I missed the social relations I have at work... “ (A3- hearing).

These experiences illustrate how digital communication can both facilitate connection and heighten emotional strain, underscoring its limits as a substitute for in-person interaction. While technology offers essential means for social participation, it cannot fully replace the relational depth and embodied presence that many participants with disabilities associate with meaningful social belonging.

## Discussion

The absence of up-to-date, gender-specific data on women with disabilities, both in periods of stability and in crisis, implies that expanding research efforts in this area could offer important insights. The present study aimed to address this issue by examining the challenges and circumstances faced by women with motor, hearing, and visual disabilities through their personal experiences and perspectives during COVID-19 and afterwards.

According to the ICF [29], participation involves integrating individuals with disabilities into the community, supporting their engagement in daily activities, and facilitating their interaction with physical and social environments [43,44].

The participants in the current study identified their bodily impairment as a central and enduring dimension of their live experience. One that shaped their sense of identity, daily functioning and interaction with the world around them. Yet, consistent with the ICF model, the study findings indicated that disability cannot be understood solely in relation to impairment. Instead, intersecting environmental, social, attitudinal, and gendered contexts played a decisive role in shaping participants’ opportunities for participation and well-being, both in times of stability and during periods of crisis. Our study shows that the pandemic magnified the interaction between environmental constraints and personal resources, illuminating how these dynamics shaped participants’ experiences of vulnerability and resilience.

Despite these intersecting challenges, participants described an overarching sense of satisfaction, agency, and resilience in their daily lives. Fulfilling employment that ensured financial independence, close-knit family relationships, and supportive community networks emerged as key protective factors. These sources of strength coexisted with and at times mitigated the ongoing challenges of living as women with disabilities, enabling participants to sustain a meaningful and engaged life.

Notably, most participants in this study identified as religious. They reported that their faith in God gave them emotional strength to cope with the challenges in daily life with a positive outlook, especially in times of crisis. This finding was consistent with Koenig et al. [45] regarding the important link between spirituality and health. A review by Zhang et al. [46] related specifically to the overall role of spiritual fortitude in mental health outcomes during crisis such as the COVID-19 pandemic. Studies show that religion and spirituality play an essential role in the lives of people with disabilities as a source of strength and support. Treloar [47] found that adults with disabilities and family members spiritual beliefs served as a stabilizing force in their lives, offering meaning to their experience of disability and supporting their coping efforts. Studies indicate that religion plays a key role in how individuals interpret and cope with chronic pain. Faith in God often provides strength and resilience in managing pain, though interpretations of suffering and its meaning tend to vary across different religious traditions [48,49]. In O’Hanlon [50] study, family members of children with special needs noted that involvement in religious activities and support from clergy and community members were meaningful, and they expressed high levels of satisfaction with both.

In line with Hansen and Philo’s proposal [51] to explore the intersection of disability and space, our findings indicated that the drastic pandemic restriction on outdoor activities increased the participants’ experience of vulnerability and threatened their independence; but it also offered advantages. The latter included less physical strain and better management of daily routines. Similar to Lindsey et al. [19], some of the participants in our study also reported that they benefitted from the disruption of their routine, by learning to appreciate life. Some participants utilized the time to implement previous plans and make significant life changes, such as pursuing further education, writing a book, or changing their profession. In these ways, the new circumstances appeared to diminish their experience of disability.

Our study showed that a variety of online technologies and services had a positive impact on women with motor, hearing, and visual disabilities experiences during and after the pandemic. In daily life, the technologies helped them overcome barriers caused by inaccessible environments. At the onset of the COVID-19 pandemic, ensuring accessible information was recognized as essential to preventing discrimination against people with disabilities in society’s response to the crisis [52].

Nonetheless, part of our study participants described the intensive use of digital technologies as enforcing strong feelings of loneliness. Although they acknowledged the advantages of these technologies, they deeply missed face-to-face interactions. Studies indicate that individuals with disabilities often feel excluded from full participation in the digital society [53]. Stough and Kang [10] showed that the decline in perceived quality of life of individuals with disabilities in the aftermath of a disaster was primarily attributed to the loss of social networks and of a sense of belonging, rather than a reduction in perceived instrumental support.

Masana and Vidal [54] revealed that individuals with “locked-in syndrome” were accustomed to a life of confinement, which enabled them to adapt more effectively to the pandemic. This was also evident in our study. The use of online technologies offered a few of our participants the potential to challenge public attitudes about disability. Specifically, women with paraplegia viewed staying at home as an opportunity to offer individuals without disabilities a glimpse into their everyday lives, potentially changing stigmas about individuals with disabilities. They mentioned that digital communication presented an opportunity to encourage more positive attitudes toward them, as only their upper body, and sometimes only above the shoulders, was visible on the screen. This finding was mentioned by others [55].

The participants in our study emphasized their independence as a central aspect of their lives, whether in making decisions, navigating public spaces, or achieving financial self-sufficiency. At the same time, they warmly described their relations with others in their community. They described the care they received daily, for example, from neighbors, relatives, and others, also during the pandemic. This included visits, phone calls, assistance with shopping and various tasks, and more. However, they also discussed their overlapping caregiving roles and responsibilities, which extended beyond personal care to include support for others both with and without disabilities. Edwards and Loughnane [56] underscored the need to reassess our understanding of care practices among individuals with disabilities. The concept of relational autonomy criticizes the independent living movement, arguing that independence and self-determination are grounded in networks of care [57]. Ignoring disabled people’s caregiving and reciprocal relationships not only undermines their agency and citizenship, but also overlooks the relational ways in which they build and sustain their lives [56].

In our study, about half of the participants were mothers, and among these mothers, about half had between four and seven children. Israel is a pro-natalist culture with a social expectation to have children. Studies show that motherhood and starting a family are among the top priorities for women in Israel [58]. Bloom et al.‘s [59] study showed that women with disabilities were just as likely as women without disabilities to express a desire to have a baby (61% vs. 60%). However, only 43% of women with disabilities intended to have a child in the future, compared to 50% of women without disabilities. This reveals a greater gap among women with disabilities between the desire for children and the intention to have them.

The participants in our study did not articulate specific concerns related to parenting in the context of their disability. They embraced parenting as a natural and meaningful part of their lives and spoke of their children as a central source of pride. However, a few mentioned the burden of finding daily activities to keep their children busy during lockdowns. Studies of the impact of COVID-19 on women confirmed that their unpaid workload increased with children being out of school [60]. In our sample, a small number also mentioned financial difficulties related to the intensive care of children who were at home during the pandemic. At the same time, they highlighted this period as quality family time.

Participants without children who returned to live with their parents during this period also appreciated this time for its emotional and often financial support, as found in Lindsay et al. [19].

### Strengths, limitations and recommendations for future studies

The importance of this research consists in giving a voice to women with disabilities during and after crisis such as the COVID-19 pandemic. The literature gives limited attention to the life experience women with disabilities, especially during times of crisis. Our sample was relatively broad, compared to typical qualitative studies [61], allowing us to gain deep insights into the perspectives of women with varied disabilities. However, the study has a few limitations. First, we conducted a convenience sample among women with motor and sensory disabilities, mostly religious and educated. Therefore, our conclusions may not reflect the experiences of women with other characteristics. Second, our analysis focused on emerging themes from the participating group, almost without exploring themes relating to specific disabilities; therefore, issues uniquely impacting each disability may have been missed. Third, data were collected after the pandemic ended, possibly missing the participants’ earliest pandemic experiences. On the other hand, this timing allowed the interviewees to reflect on the pandemic period and its impact on their lives, both positive and negative, from a perspective of time. Finally, unfortunately, none of the participants welcomed the opportunity to comment on the findings, which could have contributed to the validation of the results. However, the study included a relatively large number of participants compared to typical qualitative research [62], which may strengthen the themes we produced and the refinement of the research insights.

Future studies should expand these findings by focusing on women with a wide range of disabilities, from diverse socio-economic and ethnic groups. Examining the experiences of disabled minority women, such as Israel’s Arab women [63], is essential due to their increased vulnerability from the added intersectionality of ethnic minority status.

## Conclusions and policy implications

In keeping with the recommendation of Snæfríðar-og Gunnarsdóttir and Löve [64], integrating lived disability experiences into policy is crucial for inclusive disaster planning and crisis management. Studies have noted the need for tailored approaches to support the diverse needs of people with different disabilities during crisis, including their health care and mental health conditions [7,18,65,66]. Similarly, the UN Policy Brief on a Disability-Inclusive Response to COVID-19 [67] urges governments to embed the participation of people with disabilities at every stage of crisis planning and recovery, especially in resource limited settings. Pearce et al. [68] and Hunt et al. [69] further demonstrate that inclusive approaches grounded in community participation and local capacity building are essential for effective, equitable responses in both low- and high-income contexts.

Examining the disaster risks faced by people with disabilities also offers insights that policymakers can apply to understanding the risks faced by other groups in society [70,71]. We support Jalali et al.‘s [15] recommendation to maintain written records of the medical and rehabilitation needs of persons with disabilities, in order to ensure continuity of care during crisis. Also, proactive planning and training are essential to guarantee accessible information in future crises [72].

UN Women [22] highlights that women with disabilities are disproportionately affected in emergencies and calls for gender-responsive and disability-inclusive disaster management strategies. We recommend that policymakers adopt gender mainstreaming policies [73] in emergency programs for people with disabilities. There are issues specifically affecting women with disabilities in extreme risk situations, which also resonate significantly among women without disabilities. These include concerns about the economic situation, the increased burden in domestic workload, and worries about family members’ health [24,74,75]. We also endorse the recommendation of Aydemir-Döke et al. [24] that during extended crisis, government policymakers should enhance informal support systems for women, and specifically for women with disabilities, by increasing their access to respite care and by expanding family services.

Building on multidisciplinary rehabilitation research, our findings underscore the need for integrated psychosocial and physical support models tailored to women with disabilities during crises. Consistent with Nosek and Hughes [76], gender-specific approaches in rehabilitation are critical to addressing the compounded psychosocial vulnerabilities and structural inequities that women experience. Evidence from Óladóttir et al. [77] further highlights that rehabilitation practices often remain insufficiently gender-sensitive, with women reporting less person-centered care and fewer opportunities for family involvement, gaps that can widen during emergencies. Similarly, Abou-Abbas et al. [78] demonstrate that women and girls with disabilities in humanitarian settings face intertwined physical, psychological, and social barriers that require coordinated, community-based rehabilitation frameworks. Finally, the findings of Isiko et al. [79] emphasize the importance of embedding women lived experiences into intervention design to ensure culturally responsive psychosocial and advocacy support in resource-limited contexts.

In addition to psychosocial and physical support, attention to long-term economic recovery is essential. Crises often intensify pre-existing employment inequalities for women with disabilities, undermining their financial stability and limiting future opportunities. Feminist rehabilitation scholarship highlights that meaningful employment is central to women’s empowerment, safety, and independence, and that gender responsive vocational rehabilitation is critical for sustaining job prospects and enabling fuller participation in community life [80].

Together, these insights highlight the need for emergency preparedness and recovery strategies that combine physical rehabilitation, psychosocial support, gender responsive empowerment, and accessible employment services. Such integrated approaches are essential for strengthening economic resilience and enabling the sustained societal reintegration of women with disabilities after crises.
